# St. George's Hospital

**Published:** 1916-05-06

**Authors:** F. J. Frankau

**Affiliations:** Deputy Treasurer.


### THE EDITOR'S LETTER-BOX.

### St. George's Hospital.

*To the Editor of* The Hospital.

Sir,—I am sorry that my last letter failed to make the position clear. I think, however, the following chronological statement should do so:—

Up to May 5 we had to take overdrafts amounting to £5,300 from our bank.

On May 8 we sold Midland Stock for £12,199 to repay the bank overdraft and to meet current expenditure.

On June 1 we received under the Crokat legacy stocks valued at £8,423, which we still retain, as there was sufficient balance in hand then and has been since.

I would add that the £5,000 received on October 29 on account of the Patrickson legacy was still on deposit at the end of 1915.

I trust I have now made the position really clear.—Yours faithfully,

F. J. Frankau, Deputy Treasurer.

April 28, 1916.

[* * * We thank Mr. Frankau for his courtesy. If the paragraph which occasioned our comment had been drafted with the remembrance that the report referred to the year's income and expenditure account as a whole, and not to the state of assets and liabilities at different dates within the year, no misunderstanding could have arisen. The paragraph referred to the year's deficiency and how that deficiency had been provided for.—Ed. The Hospital.]

